# Phylogeny of Shrew- and Mole-Borne Hantaviruses in Poland and Ukraine

**DOI:** 10.3390/v15040881

**Published:** 2023-03-29

**Authors:** Fuka Kikuchi, Satoru Arai, Janusz Hejduk, Ai Hayashi, Janusz Markowski, Marcin Markowski, Leszek Rychlik, Vasyl Khodzinskyi, Hajime Kamiya, Tetsuya Mizutani, Motoi Suzuki, Beata Sikorska, Paweł P. Liberski, Richard Yanagihara

**Affiliations:** 1 Center for Surveillance, Immunization and Epidemiologic Research, National Institute of Infectious Diseases, Tokyo 162-8640, Japan; 2 Center for Infectious Diseases Epidemiology and Prevention Research, Tokyo University of Agriculture and Technology, Tokyo 183-8509, Japan; 3 Department of Biodiversity Studies and Bioeducation, Faculty of Biology and Environmental Protection, University of Łódź, 90-237 Łódź, Poland; 4 Tohoku University Graduate School of Medicine, Sendai 980-8575, Japan; 5 Department of Experimental Zoology and Evolutionary Biology, Faculty of Biology and Environmental Protection, University of Łódź, 90-237 Łódź, Poland; 6 Department of Systematic Zoology, Institute of Environmental Biology, Faculty of Biology, Adam Mickiewicz University, 61-614 Poznań, Poland; 7 Institute of Forestry and Park Gardening, Ukrainian National Forestry University, 79057 Lviv, Ukraine; 8 Department of Molecular Pathology and Neuropathology, Medical University of Łódź, 92-216 Łódź, Poland; 9 Departments of Pediatrics and Tropical Medicine, Medical Microbiology and Pharmacology, John A. Burns School of Medicine, University of Hawaii at Manoa, Honolulu, HI 96813, USA

**Keywords:** *Hantaviridae*, hantavirus, shrew, mole, Poland, Ukraine

## Abstract

Earlier, we demonstrated the co-circulation of genetically distinct non-rodent-borne hantaviruses, including Boginia virus (BOGV) in the Eurasian water shrew (*Neomys fodiens*), Seewis virus (SWSV) in the Eurasian common shrew (*Sorex araneus*) and Nova virus (NVAV) in the European mole (*Talpa europaea*), in central Poland. To further investigate the phylogeny of hantaviruses harbored by soricid and talpid reservoir hosts, we analyzed RNAlater^®^-preserved lung tissues from 320 shrews and 26 moles, both captured during 1990–2017 across Poland, and 10 European moles from Ukraine for hantavirus RNA through RT-PCR and DNA sequencing. SWSV and Altai virus (ALTV) were detected in *Sorex araneus* and *Sorex minutus* in Boginia and the Białowieża Forest, respectively, and NVAV was detected in *Talpa europaea* in Huta Dłutowska, Poland, and in Lviv, Ukraine. Phylogenetic analyses using maximum-likelihood and Bayesian methods showed geography-specific lineages of SWSV in Poland and elsewhere in Eurasia and of NVAV in Poland and Ukraine. The ATLV strain in *Sorex minutus* from the Białowieża Forest on the Polish–Belarusian border was distantly related to the ATLV strain previously reported in *Sorex minutus* from Chmiel in southeastern Poland. Overall, the gene phylogenies found support long-standing host-specific adaptation.

## 1. Introduction

Formerly grouped in the family *Bunyaviridae* and the genus *Hantavirus*, hantaviruses were recently reclassified into a new order (Bunyavirales) and family (*Hantaviridae*), the latter comprising four subfamilies (*Actantavirinae*, *Agantavirinae*, *Mammantavirinae* and *Repantavirinae*) [1,2]. The subfamily *Mammantavirinae* consists of four genera (*Loanvirus*, *Mobatvirus*, *Orthohantavirus* and *Thottimvirus*) based on DivErsity pArtitioning by hieRarchical Clustering (DEmARC) analysis and using concatenated complete S and M amino acid-coding regions [1]. Hantaviruses possess a single-stranded, negative-sense RNA genome consisting of three segments designated large (L), medium (M) and small (S), which encode an RNA-dependent RNA polymerase (RdRp), envelope glycoproteins (Gn and Gc) and a nucleocapsid (N) protein, respectively [3,4].

Members of the family *Hantaviridae* have no known arthropod hosts. Viruses of the subfamilies *Actantavirinae* and *Agantavirinae* are found in fish, whereas those of the subfamily *Repantavirinae* are hosted by reptiles. By contrast, viruses of the subfamily *Mammantavirinae* are harbored by small mammals belonging to three taxonomic orders: Rodentia, Eulipotyphla and Chiroptera [5,6]. Several hantaviruses hosted by rodents of the families Muridae and Cricetidae in Eurasia and the Americas cause diseases of varying clinical severity in humans; these are known as hemorrhagic fever with renal syndrome (HFRS) [7,8,9] and hantavirus cardiopulmonary syndrome (HCPS) [10,11].

All rodent-borne hantaviruses belong to the genus *Orthohantavirus*, which also includes nearly all recently identified hantaviruses carried by multiple species of shrews [5,6]. By contrast, mole-borne hantaviruses group with orthohantaviruses, thottimviruses and mobatviruses, and thus, they appear to be more permissive in their host proclivity, suggesting that ancestral moles may have served as the early hosts of primordial hantaviruses [5,12,13]. However, the recent discovery of hantaviruses in bats has further enriched discussions about the evolutionary history of hantaviruses [6]. To date, hantaviruses carried by bats of the suborders Yinpterochiroptera and Yangochiroptera are restricted to the genera *Loanvirus* and *Mobatvirus* [6,14,15]. All but one of the 13 bat-borne hantaviruses reported thus far have been from Asia and Africa. The exception is Brno loanvirus, originally detected in the common noctule (*Nyctalus noctula*) in the Czech Republic [16]. A recent study showed Brno loanvirus in the visceral tissues of common noctules in Austria, Germany and Poland [17].

Guided by studies reporting HFRS antigens in the Eurasian common shrew (*Sorex araneus*), Eurasian pygmy shrew (*Sorex minutus*), Eurasian water shrew (*Neomys fodiens*) and European mole (*Talpa europaea*) in Russia and the former Yugoslavia nearly four decades ago [18,19,20], and by gaining access to the treasure trove of archival tissues in museums and private collections, the molecular detection and subsequent genomic characterization of phylogenetically distinct hantaviruses across space and time accelerated in shrews and moles (order Eulipotyphla, families Soricidae and Talpidae) from Eurasia [5,6,21]. An initial exploratory study in Poland demonstrated a novel hantavirus, designated Boginia virus (BOGV), in the Eurasian water shrew in Boginia, Huta Dłutowska and Kurowice as well as Seewis virus (SWSV) in the Eurasian common shrew in Boginia and Huta Dłutowska [22]. More recently, we reported the co-circulation of SWSV in the Eurasian common shrew and Mediterranean water shrew (*Neomys anomalus*) in Boginia, of BOGV and SWSV in Kurowice and of BOGV, SWSV and Nova virus (NVAV) in the European mole in Huta Dłutowska with no evidence of spill-over infection from shrews to moles or vice versa [23]. Moreover, we identified Altai virus (ATLV) in a European pygmy shrew from Chmiel in southeastern Poland [24] and SWSV in Eurasian common shrews from Osobowice in southwestern Poland [25]. However, we failed to detect hantaviruses in RNAlater^®^-preserved patagia and feces from vesper bats in Poland [26].

To better understand the phylogeny of soricid- and talpid-borne hantaviruses in Poland, we analyzed archival tissues from shrews and moles captured across Poland in 1999–2017 as well as European moles from Ukraine captured near the Polish border in 2016. The high prevalence of NVAV in European moles in Poland was not unexpected, but what was noteworthy was the detection of NVAV in European moles from Ukraine. Although reports appeared recently about rodent-borne hantaviruses in Ukraine [27,28], specifically Dobrava virus/Belgrade virus (DOBV/BGDV) in the striped field mouse (*Apodemus agrarius*) and yellow-necked mouse (*Apodemus flavicollis*) and Puumala virus (PUUV) in the bank vole (*Myodes glareolus*), there have been no publications to our knowledge on non-rodent-borne hantaviruses.

## 2. Materials and Methods

### 2.1. Trap Sites and Specimen Processing

**Figure 1:**
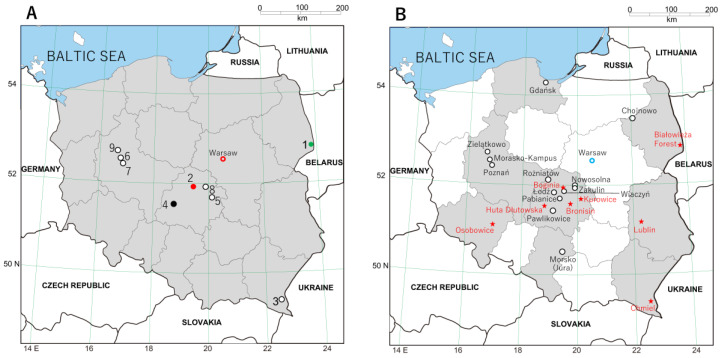
(**A**) Map of Poland showing the nine sites where shrews and moles were captured in the present study: (1) Białowieża Forest; (2) Boginia; (3) Chmiel; (4) Huta Dłutowska; (5) Kurowice; (6) Poznań, Morasko-Kampus; (7) Poznań, city center; (8) Zakulin; (9) Zielątkowo. Hantaviruses are color-coded: ALTV (green circle); SWSV (red circle); NVAV (black circle); hantavirus RNA not detected (white circle). (**B**) Map of Poland showing the 21 trap sites in eight provinces where shrews and moles were captured in all published studies. Red stars indicate the eight sites where hantavirus-infected shrews and/or moles were found. White circles indicate the 13 sites where hantavirus RNA was not detected in shrews and moles.

Shrews and moles, captured during 1990–2017 in nine sites across Poland (Figure 1A) as previously described [22,23], were euthanized by means of cervical dislocation. Carcasses were then stored at –20 °C for months to years before lung tissues were dissected and preserved in RNAlater^®^ RNA Stabilization Reagent (Qiagen, Valencia, CA, USA). In earlier studies, shrews and moles were collected in Boginia, Chmiel, Huta Dłutowska and Kurowice [22,23]. However, the Global Positioning System coordinates of the trap sites in these sites were different in the present study: Boginia (51°50′18.05″ N, 19°36′41.85″ E), Huta Dłutowska (51°35′41.69″ N, 19°23′2.63″ E) and Kurowice (51°39′57.90″ N, 19°42′8.95″ E) in Łódź province; Chmiel (49°13′14.80″ N, 22°36′8.12″ E) in Subcarpathia province. Newly sampled sites included Białowieża Forest (52°43′6.02″ N, 23°51′0.21″ E) in Podlasie province; Poznań, Morasko-Kampus (52°28′1.67″ N, 16°55′22.51″ E), Poznań, city center (52°24′22.55″ N, 16°56′4.44″ E) and Zielątkowo (52°33′29.13″ N, 16°47′33.82″ E) in Greater Poland province; Zakulin (51°59′31.48″ N, 19°56′19.53″ E) in Łódź province. In addition, lung tissues from European moles captured in Lviv (49°50’16.93″ N, 24°0’29.64″ E) in Ukraine in 2016 were analyzed (Table 1).

### 2.2. Ethics Statement

All trapping and experimental procedures on shrews and moles from Poland were approved by the Łódź Ethical Committee on Animal Testing (14/LB/511/2010 and 29/LB/548/2011), the General Directorate for Environmental Protection (DOP-OZGiZ.4200/N2732/10/JRO, DOP-OZGiZ.6401.05.25.2011kp.3 and DOP-OZGiZ.6401.05.28. 2011kp.1) and the Łódź Regional Directorate for Environmental Protection (WPN.6401.1012017.ŁL.Z and WPN.6401.06.2017.T.Dz). For shrews captured in Białowieża Forest, approvals were obtained from the Local Ethical Committee for Animal Experiments in Białystok (2005/46), the Local Ethical Committee for Animal Experiments in Poznań (10/2007) and the General Directorate for Environmental Protection (DOPog-4201-04-126/05/kl and DLOPiK-op/ogiz-4200/IV-7/1331/08/aj). For shrews captured in Poznań and Zielątkowo, approvals were obtained from the Local Ethical Committee for Animal Experiments in Poznań (61/2012 and 14/2015) and the Regional Directorate for Environmental Protection in Poznań (WPN-II.6401.142.2013.AG and WPN-II.6401.249.2017.AC). For moles captured in Ukraine, approval was provided by the Institute of Forestry and Park Gardening of the Ukrainian National Forestry University.

### 2.3. RNA Extraction, cDNA Synthesis and RT-PCR Amplification

RNAlater^®^-preserved lung tissues from shrews and moles were analyzed for hantavirus RNA using nested or hemi-nested RT-PCR and using oligonucleotide primers and protocols that previously resulted in the successful amplification of hantavirus RNA in tissues of shews, moles and bats [14,15,22,23,29]. Total RNA was extracted using the MagDEA RNA100 Kit (Precision System Science, Matstudo, Japan), and cDNA was synthesized using the PrimeScript II 1st strand cDNA Synthesis Kit (Takara Bio, Inc., Otsu, Japan) with oligonucleotide primer (OSM55F, 5′-TAGTAGTAGACTCC-3′) that was designed from conserved 5′-ends of the S, M and L segments of hantaviruses [30]. First- and second-round PCR trials were performed in 20-μL reaction mixtures containing 250 μM dNTP, 2.5 mM MgCl2, 1 U Takara LA Taq polymerase Host Start version (Takara Bio, Inc.) and 0.25 μM of each primer [31]. The oligonucleotide primer sequences used for nested or hemi-nested PCR trials were OSM55 and HTN-S6 (5′-AGCTCNGGATCCATNTCATC-3′), followed by Cro-2F (5′-AGYCCNGTNATGRGWGTNRTYGG-3′) and Cro-2R (5′-ANAYTGRTARAANGANGAYTTYTT-3’) for the S segment; OSV697F (5′-GGACCAGGTGCADCTTGTGAAGC-3′) and TM1485R (5’-CCAGCCAAARCARAATGT-3’), and then TM1199F (5’-TAAVTTCAMCAACATGTCT-3’) and TM1485R for the M segment; HAN-L-F1 (5′-ATGTAYGTBAGTGCWGATGC-3′), and HAN-L-R1 (5′-AACCADTCWGTYCCRTCATC-3′), and then HAN-L-F2 (5′-TGCWGATGCHACNAARTGGTC-3′) and HAN-L-R2 (5′-GCRTCRTCWGARTGRTGDGCAA-3′) for the L segment. Initial denaturation at 94 °C for 2 min was followed by two cycles each of denaturation at 94 °C for 30 s, two-degree step-down annealing from 46 °C to 38 °C for 40 s and elongation at 72 °C for 1 min, then 30 cycles of denaturation at 94 °C for 30 s, annealing at 42 °C for 40 s and elongation at 72 °C for 1 min in a Veriti thermal cycler (Applied Biosystems, Foster City, CA, USA) [14,15].

### 2.4. Genetic and Phylogenetic Analyses

Partial S- and L-segment nucleotide sequences were aligned with SWSV, NVAV and other representative hantavirus sequences available on GenBank using the ClustalW in BioEdit [32]. The degree of sequence homology was assessed using pairwise comparisons [13,30]. Phylogenetic trees were constructed using the maximum-likelihood method implemented in PAUP* (Phylogenetic Analysis Using Parsimony, version 4.0a169) and MrBayes 3.1.2 [33] under the GTR+I+Γ model of evolution, as selected by using jModelTest version 2.1.7 [34]. Bayesian analysis consisted of 10 million Markov chain Monte Carlo generations to ensure convergence across two runs of six chains each, with average standard deviations of split frequencies less than 0.01 and effective sample sizes well over 100, resulting in consensus trees supported by posterior-node probabilities [13,30]. Each genomic segment was treated separately in phylogenetic analyses. The posterior node probabilities were based on two million generations and estimated sample sizes over 100 (implemented in MrBayes). Maximum-likelihood tress were evaluated via bootstrap analysis of 100 iterations, which was implemented in PAUP*.

## 3. Results

In analyzing RNAlater^®^-preserved archival lung tissues from 320 shrews (117 Eurasian common shrews, 66 Eurasian pygmy shrews, 65 Mediterranean water shrews and 72 Eurasian water shrews) captured at multiple sites in Poland (Figure 1A), we detected hantavirus RNA in one Eurasian common shrew from Boginia and one Eurasian pygmy shrew from Białowieża Forest (Table 1). By contrast, of the 26 European moles from Poland and 10 from Ukraine, hantavirus RNA was detected via nested RT-PCR in eight and two of them, respectively. The identities of the hantaviruses were determined via DNA sequencing and genetic and phylogenetic analysis.

Pairwise alignment and comparison of partial S- and/or L-segment sequences demonstrated ALTV strain PL7814LR56 in a Eurasian pygmy shrew from Białowieża Forest, SWSV strain PL7663JH151 in a Eurasian common shrew from Boginia and NVAV strains in European moles from Huta Dłutowska (Poland) and Lviv (Ukraine). The partial 347-nucleotide L-segment sequence of SWSV strain PL7663JH151 was >98% and 100% similar at the nucleotide and amino acid level, respectively, to SWSV strains 1107 and 3334, which was previously reported from Boginia and Kurowice (Supplementary Table S1). Analyses of the partial 353-nucleotide L-segment sequences of the NVAV strains showed uniformly high similarities within strains from Poland and the two strains from Ukraine (PL7965LW001TA and PL7970LW006TA), with divergences of approximately 15% at the nucleotide level and <5% at the amino acid level between NVAV strains from both countries (Supplementary Table S1).

The 707- and 698-nucleotide S-segment sequences of NVAV strain PL7712JH226 from Poland and of NVAV strain PL7970LW006TA from Ukraine, respectively, showed 84% and 91% sequence similarity at the nucleotide and amino acid levels, respectively. Despite repeated attempts, the M-segment primers yielded no amplicons.

**Figure 2:**
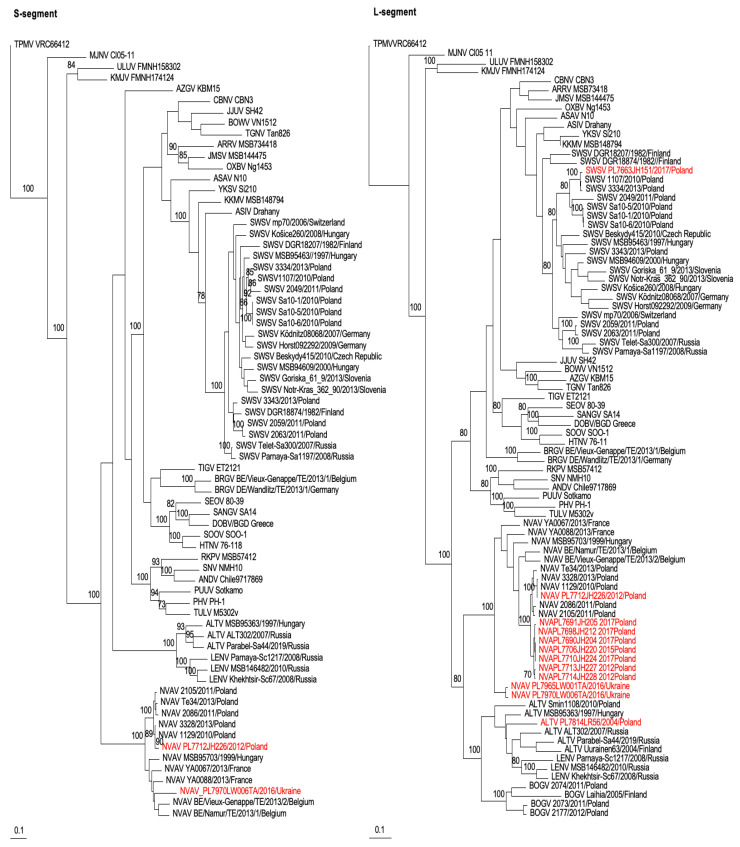
Phylogenetic trees generated by way of the maximum-likelihood method using PAUP* and based on the 698- and 707-nucleotide S-segment and the partial 353-nucleotide L-segment sequences of NVAV from European moles captured in Poland, Ukraine, Belgium, France and Hungary, showing geographic-specific clustering. Similarly, SWSV strains from Poland and elsewhere are grouped according to geography. Newfound sequences from Poland and Ukraine are shown in red lettering. SWSV, ALTV and NVAV strains show the year of capture of the host and the country of origin. GenBank numbers for all taxa are provided in Supplementary Table S2. The numbers at selected nodes are bootstrap support values of >70 (expressed as the percentage of replicates in which the node was recovered), which were determined for 100 maximum-likelihood iterations under the same model of evolution using PAUP* version 4.0a169 (http://phylosolutions.com/paup-test/, accessed on 15 February 2023). The scale bar indicates 0.1 nucleotide substitutions per site.

**Figure 3:**
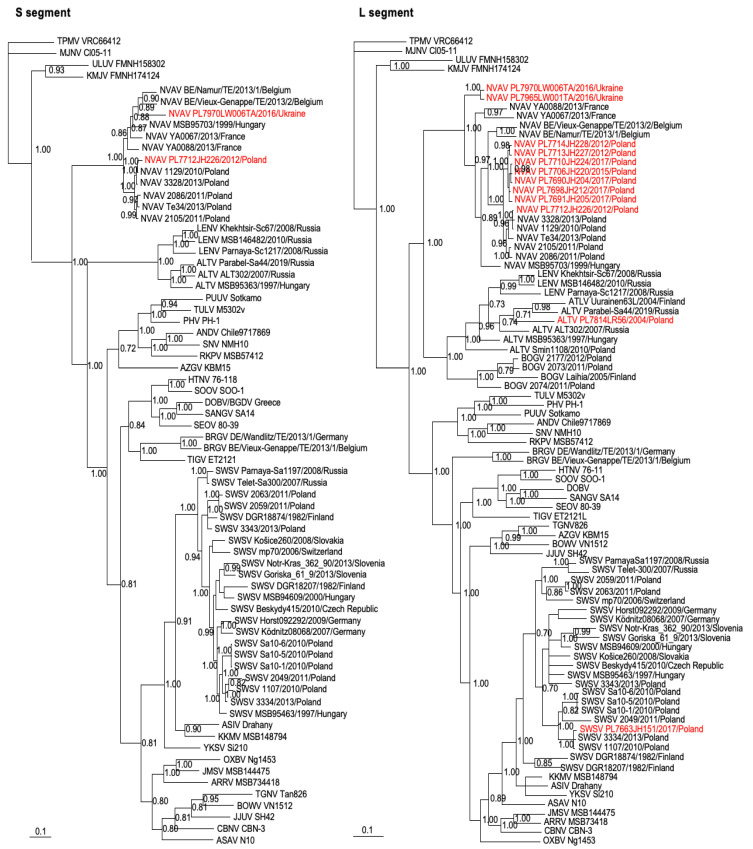
Phylogenetic trees generated by the Bayesian method, under the best-fit GTR+I+Γ model of evolution, based on the 698- and 707-nucleotide S-segment and the partial 353-nucleotide L-segment sequences of NVAV from European moles captured in Poland, Ukraine, Belgium, France and Hungary, showing geographic-specific clustering. Similarly, SWSV strains from Poland and elsewhere are grouped according to geography. Newfound sequences from Poland and Ukraine are shown in red lettering. SWSV, ALTV and NVAV strains show the year of capture of the host and the country of origin. GenBank numbers for all taxa are provided in Supplementary Table S2. The numbers at selected nodes are Bayesian posterior probabilities > 0.7 based on 150,000 trees; two replicate Markov chain Monte Carlo runs, consisting of six chains of 10 million generations, each sampled every 100 generations with a burn-in of 25,000 (25%). Scale bars indicate 0.1 nucleotide substitutions per site.

Phylogenetic analyses, performed by way of the maximum-likelihood method using PAUP*, showed well-supported trees (Figure 2). Similarly, Bayesian methods showed equally robust trees with a tendency toward geographic-specific clustering (Figure 3 and Supplementary Figure S1). Specifically, analyses of partial S- and L-segment sequences of SWSV indicated clustering of strains from Poland (Figure 2 and Figure 3), whereas ALTV strain PL7814LR56 from Białowieża Forest in northeastern Poland was more distantly related to ALTV strain Smin1108 from Chmiel in southeastern Poland. This may be attributed to the non-overlapping short sequences between the two ALTV strains.

The GenBank accession numbers for the newfound hantavirus sequences include SWSV PL7663JH151 (L: OQ341654) from *Sorex araneus* captured in Boginia, Poland; ATLV PL7814LR56 (L: OQ341663) from *Sorex minutus* captured in Białowieża Forest, Poland; NVAV PL7690JH204 (L: OQ341655), NVAV PL7691JH205 (L: OQ341656), NVAV PL7698JH212 (L: OQ341657), NVAV PL7706JH220 (L: OQ341658), NVAV PL7710JH224 (L: OQ341659), NVAV PL7712JH226 (S: OQ352297; L: OQ341660), NVAV PL7713JH227 (L: OQ341661) and NVAV PL7714JH228 (L: OQ341662) from *Talpa europaea* captured in Huta Dłutowska, Poland; NVAV PL7965LW001TA (L: OQ341664) and NVAV PL7970LW006TA (S: OQ352298; L: OQ341665) from *Talpa europaea* captured in Lviv, Ukraine.

In addition, phylogenetic analyses of NVAV strains from Poland and Ukraine, including prototype NVAV from Hungary (MSB95703) and representative NVAV strains from Poland (1129, 2086, 2105, 3328, Te34), France (YA0067 and YA0088) and Belgium (Namur, Vieux-Genappe), showed segregation according to geography, despite the short sequences. The tree topologies were well-supported, with posterior node probabilities of >0.9 for the major rodent-, shrew- and mole-borne hantavirus species (Figure 3).

Table 2 summarizes the geographic distribution of ALTV, BOGV, SWSV and NVAV in Poland based on findings from the present study and those of previous published studies. Hantavirus RNA was detected in 20 of 206 *Sorex araneus*, 5 of 134 *Sorex minutus*, 1 of 77 *Neomys anomalus*, 7 of 94 *Neomys fodiens* and 47 of 95 *Talpa europaea* captured in 8 of 21 sites in Poland (Figure 1B). However, some trap sites were insufficiently sampled. For example, only one *Sorex araneus* was captured in Morkso (Jura), Nowosolna and Pabianice, and only two were captured in Gdańsk; only one *Talpa europaea* was captured in Kurowice, Łódź, Pawlikowice, Rożniatów, Wiączyń and Zakulin.

## 4. Discussion

Previously, we reported BOGV in Eurasian water shrews and SWSV in Eurasian common shrews and Eurasian pygmy shrews from central Poland [22]. Subsequently, we reported the co-circulation of BOGV, SWSV and NVAV in Boginia, Kurowice and Huta Dłutowska [23]. In this study, we failed to detect hantavirus RNA in most of the archival shrew tissues. Because tissues were harvested from whole carcasses stored for prolonged periods under suboptimal temperatures, poorly preserved or degraded RNA may have been contributory. Nevertheless, finding an ATLV strain in a Eurasian pygmy shrew captured in Białowieża Forest in 2004 is significant, as it represents only the second observation of such to date. Located adjacent to the border between Poland and Belarus, Białowieża Forest, particularly the Bialowieza National Park, represents the best preserved remnants of a primeval lowland mixed forest in Europe [35], retaining its primary landscape and striking biological diversity [36]. Białowieża Forest in eastern Poland is more than 400 km from Chmiel in southeastern Poland, where the previous ALTV strain (Smin1108) was detected in a Eurasian pygmy shrew captured in 2010 [24].

The detection of NVAV in European moles from Ukraine is not unexpected, as a greater than 50% prevalence of NVAV infection was reported previously in European moles in Poland [23], France [37] and Belgium [38]. The high prevalence of NVAV infection in European moles suggests efficient virus transmission and a well-established reservoir host–hantavirus relationship similar to the high prevalence of SWSV infection in Eurasian common shrews [29]. To what extent the subterranean lifestyle of European moles also facilitates NVAV transmission is unknown, but NVAV is likely to be widespread throughout the vast geographic range of the European mole, which extends from the United Kingdom to Russia. Studies are warranted to further clarify the genetic diversity and phylogeography of NVAV. In addition, the recent discovery of Academ virus in the Siberian mole (*Talpa altaica*) in Russia [12] and Asturias virus in the Iberian mole (*Talpa occidentalis*) in Spain (S.H. Gu ad R. Yanagihara, unpublished observations) would predict that other genetically distinct hantaviruses are harbored by mole species belonging to the genus *Talpa*. Finally, studies are warranted to map the geographic distribution and genetic diversity of other non-rodent-borne hantaviruses in neighboring Ukraine and Belarus. At a minimum, the geographic range of the Eurasian common shrew, Eurasian water shrew and Eurasian pygmy shrew would predict the circulation of SWSV, BOGV and Assikala virus [39], respectively.

The pathogenic potential of NVAV and other non-rodent-borne hantaviruses to cause infection and/or disease in humans is unknown. We previously demonstrated widespread distribution of NVAV RNA in tissues of European moles [23], which is similar to that found in reservoir rodents naturally or experimentally infected with Hantaan virus [40,41] or PUUV [42]. Specifically, the demonstration of NVAV RNA in the kidneys and intestines of wild-caught European moles is consistent with viral shedding in urine and feces. However, there is no evidence of NVAV infection or disease in humans. Nevertheless, heightened awareness about the widespread distribution of NVAV and the occasional contact between humans and moles should prompt physicians to be vigilant for febrile illnesses or unusual clinical syndromes occurring among gardeners, farmers, mole catchers and field biologists, all of whom may be exposed to secretions and/or excretions of European moles or other species of moles known to harbor hantaviruses, such as the Siberian mole [12], Eastern mole (*Scalopus aquaticus*) [13], American shrew mole (*Neurotrichus gibbsii*) [43], Japanese shrew mole (*Urotrichus talpoides*) [44] and long-tailed mole (*Scaptonyx fusicaudus*) [45].

Studies showed the high prevalence of PUUV and DOBV/BGDV in reservoir rodent hosts in several regions of Poland [25,46,47,48]. Recently, a highly divergent lineage of PUUV was demonstrated in bank voles from southern Poland [49], and the Dobrava genotype of DOBV/BGDV was isolated from yellow-necked mice captured in the Subcarpathian region [50]. Moreover, molecular evidence of the Kurkino genotype of DOBV/BGDV was found in striped field mice from Lower Silesia in southwestern Poland [25]. Despite mounting evidence of the widespread distribution of disease-causing, rodent-borne hantaviruses in Poland, HFRS is reported infrequently [51,52,53,54,55,56,57]. It is generally agreed, however, that the Subcarpathia province in the southeastern part of Poland, which borders Ukraine, is an endemic area of HFRS [56,57]. Apart from disease, epidemiological surveys show serological evidence of hantavirus infection among mammalogists [58], forestry workers [59] and hunters [60]. It is not clear if HFRS cases are being misdiagnosed and/or being underreported because of insufficient clinician awareness or of limited access to standardized serodiagnostic tests.

As noted in previous reviews of this paradigm-shifting, medically important group of viruses [5,6], as well as in a recent historical account of *Hantaviridae* [61], much remains unknown, and future research is needed on many levels. For example, none of the 13 bat-borne loanviruses and mobatviruses have been isolated in cell culture, and except for Thottapalayam virus [62], Imjin virus [63] and NVAV [64], all of the more than 20 recently described hantaviruses harbored by shrews and moles exist only as partial or full-length sequences. In this regard, in continuing efforts to establish a more rigorous taxonomy of the family *Hantaviridae*, a proposal was made to require full-length S-, M- and L-segment sequences of all hantaviruses [65]. This will present obvious challenges not only for the dozens of well-known “classic” rodent-borne orthohantaviruses that have yet to be fully sequenced [65], but also for the partial hantaviral S-, M- and/or L-segment sequences detected in archival shrew, mole and bat tissues obtained from museums and field collections. Thus, for many of the non-rodent-borne putative hantaviruses which have yet to be fully sequenced, this will necessitate at least one but more probably repeated trapping expeditions for specific reservoir host species for molecular screening and subsequent whole-genome sequencing. As an obvious case in point, whole genome sequences are currently unavailable for SWSV and BOGV; thus, they would not be considered bona fide hantaviruses until each of their three segments are fully sequenced.

## Figures and Tables

**Table 1:** RT-PCR analysis for hantavirus RNA in tissues of shrews and moles from Poland and Ukraine.

Species	Country	Capture Site	Year(s)	Tested	Positive	Hantavirus
*Sorex araneus*	Poland	Białowieża Forest	1990–2005	29	0	
		Boginia	2017	14	1	SWSV
		Chmiel	2017	18	0	
		Kurowice	2017	25	0	
		Poznań (Morasko-Kampus)	2017	7	0	
		Poznań (city center)	2013–2016	24	0	
*Sorex minutus*	Poland	Białowieża Forest	1990–2004	26	1	ALTV
		Boginia	2017	18	0	
		Chmiel	2017	9	0	
		Kurowice	2017	13	0	
*Neomys anomalus*	Poland	Białowieża Forest	1992–2009	58	0	
		Chmiel	2017	7	0	
*Neomys fodiens*	Poland	Białowieża Forest	1999–2009	29	0	
		Boginia	2017	2	0	
		Chmiel	2017	1	0	
		Kurowice	2017	13	0	
		Poznań (Morasko-Kampus)	2013–2016	14	0	
		Zielątkowo	2017	13	0	
*Talpa europaea*	Poland	Huta Dłutowska	2012–2017	24	8	NVAV
		Kurowice	2017	1	0	
		Zakulin	2017	1	0	
	Ukraine	Lviv	2016	10	2	NVAV

**Table 2:** Summary of RT-PCR analysis for hantavirus RNA in shrews and moles from Poland.

Capture Site	Province	*Sorex* *araneus*	*Sorex* *minutus*	*Neomys anomalus*	*Neomys fodiens*	*Talpa* *europaea*
Białowieża Forest	Podlasie	0/29	1/26	0/58	0/29	
Boginia	Łódź	3/33	0/24		2/4	0/4
Bronisin	Łódź					1/3
Chmiel	Subcarpathia	6/36	1/30	1/19	0/1	
Chojnowo	Podlasie				0/1	
Gdańsk	Pomerania	0/2				
Huta Dłutowska	Łódź	4/18	0/9		2/2	46/82
Morsko (Jura)	Silesia	0/1				
Kurowice	Łódź	3/38	3/40		3/29	0/1
Łódź	Łódź					0/1
Lublin	Lublin	1/8				
Nowosolna	Łódź	0/1	0/5			
Osobowice	Lower Silesia	3/8				
Pabianice	Łódź	0/1			0/1	
Pawlikowice	Łódź					0/1
Poznań (city center)	Greater Poland	0/24			0/14	
Poznań (Morasko-Kampus)	Greater Poland	0/7				
Rożniatów	Łódź					0/1
Wiączyń	Łódź					0/1
Zakulin	Łódź					0/1
Zielątkowo	Greater Poland				0/13	
Hantavirus		SWSV	SWSV ATLV	SWSV	BOGV	NVAV

## Data Availability

GenBank accession numbers for phylogenetic analyses are available in Supplementary Table S1. Other presented data are available on request from the corresponding author.

## Supplementary Materials

The following supporting information can be downloaded at: https://www.mdpi.com/article/10.3390/v15040881/s1, Figure S1: Phylogenetic trees of newfound SWSV, NVAV and ALTV/LENV from Poland and Ukraine; Table S1: Pairwise Comparison of L-Segment Nucleotide Sequences of SWSV Strains; Table S2: GenBank Accession Numbers of Taxa for S and L trees **.**
