# Supplementary material for: Phylogeny of Shrew- and Mole-Borne Hantaviruses in Poland and Ukraine
Source: Viruses. 2023 Mar 29;15(4):881. doi: 10.3390/v15040881 (PMC10145205; doi:10.3390/v15040881)
Supplement: Supplementary file 1 [file viruses-15-00881-s001.zip › Figure S1.pdf]

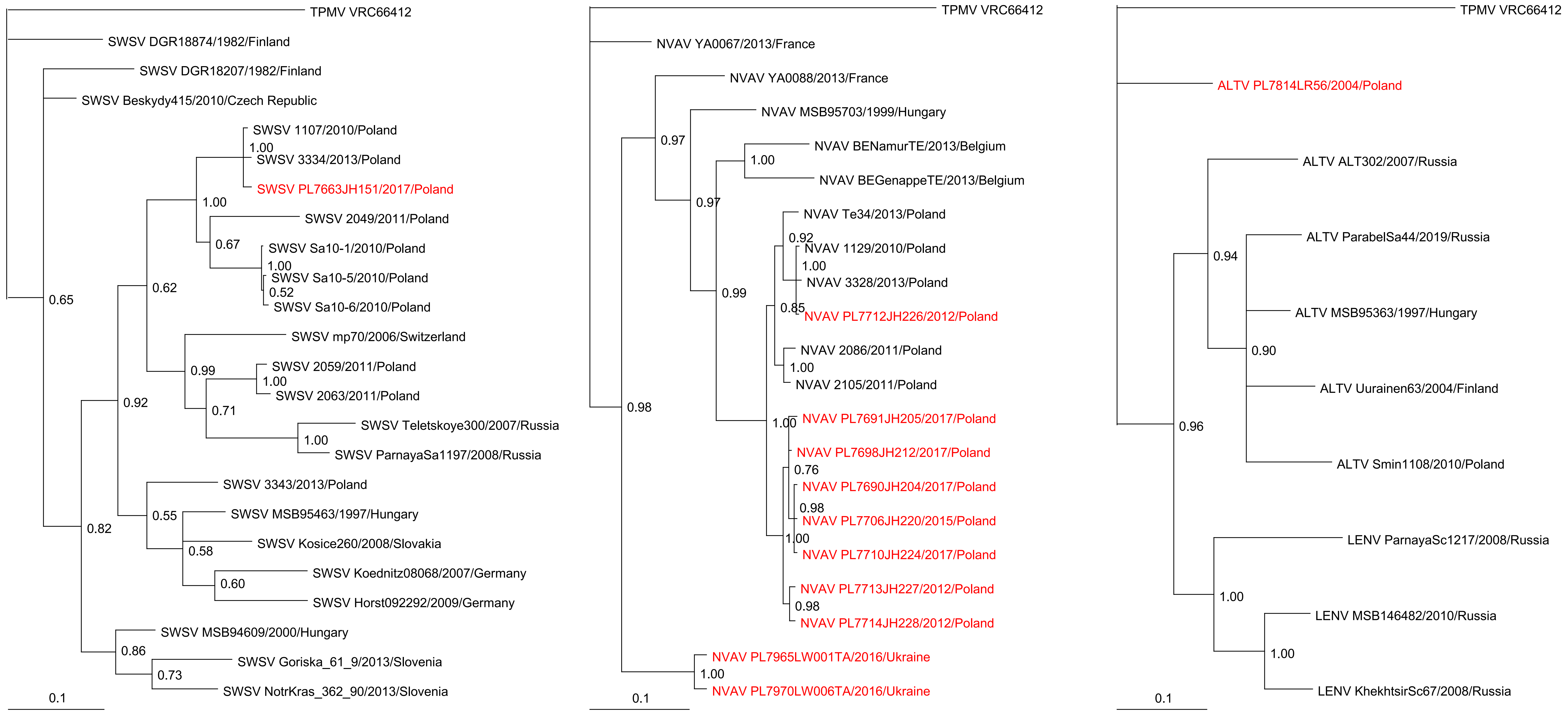

Supplementary Figure S1. Phylogenetic trees generated by the Bayesian method, under the best-fit GTR+I+Γ model of evolution, based on the partial 353-nucleotide L-genomic segment sequences of newfound SWSV, NVAV and ALTV/LENV from Poland and Ukraine, in relationship to representative SWSV, NVAV and ALTV/LENV shown in Supplemental Table S2. Newfound sequences from Poland and Ukraine are shown in red lettering. SWSV, ALTV and NVAV strains show the year of capture of the host and the country of origin. GenBank numbers for all taxa are provided in Supplementary Table S2. The numbers at selected nodes are Bayesian posterior probabilities based on 150,000 trees: two replicate Markov chain Monte Carlo runs, consisting of six chains of 10 million generations, each sampled every 100 generations with a burn-in of 25,000 (25%). Scale bars indicate nucleotide substitutions per site.
