# Supplementary material for: Phylogeny of Shrew- and Mole-Borne Hantaviruses in Poland and Ukraine
Source: Viruses. 2023 Mar 29;15(4):881. doi: 10.3390/v15040881 (PMC10145205; doi:10.3390/v15040881)
Supplement: Supplementary file 1 [file viruses-15-00881-s001.zip › Table S1.pdf]

Supplemental Table S1. Pairwise Comparison of L-Segment Nucleotide Sequences of SWSV Strains

Amino Acid Sequence Similarity

Nucleotide Sequence Similarity

	SWS 1107 2010 Poland	SWS 2059 2011 Poland	SWS 2063 2011 Poland	SWS 2049 2011 Poland	SWS 3334 2013 Poland	SWS 3343 2013 Poland	SWS Sa10-1 2010 Poland	SWS Sa10-5 2010 Poland	SWS Sa10-6 2010 Poland	**SWS PL7663JH151 2017 Poland**	SWSV mp70 2006 Switzerland	SWS MSB94609 2000 Hungary	SWS MSB95463 1997 Hungary	SWS DGR18874 1982 Finland	SWS DGR18207 1982 Finland	SWS Beskydy415 2010 Czech	SWS Kosice260 2008 Slovakia	SWS Koednitz08068 2007 Germany	SWS Horst092292 2009 Germany	SWS Goriska6193 2013 Slovenia	SWS NotrKras36290 2013 Slovenia	SWS Telet300 2007 Russia	SWS ParnayaSa1197 2008 Russia
SWS 1107 2010 Poland		0.9846	0.9576	1	1	0.9652	1	1	1	**1**	0.9925	0.9813	1	0.9851	0.9779	0.9909	0.9727	0.9703	0.9636	0.9789	0.9583	0.9652	0.9652
SWS 2059 2011 Poland	0.8107		0.9831	0.9661	1	1	0.9844	0.9844	0.9844	**0.9652**	0.9923	0.9808	1	0.9851	0.9779	0.9727	0.9727	0.9703	0.9818	0.9789	0.9792	1	1
SWS 2063 2011 Poland	0.8006	0.9803		0.9576	ND	0.9826	0.9565	0.9565	0.9565	**0.9565**	0.9746	0.9492	ND	ND	ND	0.9537	0.9537	0.9505	0.9630	0.9579	0.9583	0.9826	0.9826
SWS 2049 2011 Poland	0.8596	0.7978	0.7949		ND	0.9652	1	1	1	**1**	0.9831	0.9661	ND	ND	ND	0.9907	0.9722	0.9703	0.9630	0.9789	0.9583	0.9652	0.9652
SWS 3334 2013 Poland	0.9898	0.8041	ND	ND		ND	1	1	1	**ND**	1	0.9924	1	0.9847	0.9771	ND	ND	ND	ND	ND	ND	ND	ND
SWS 3343 2013 Poland	0.8266	0.8584	0.8555	0.8092	ND		0.9652	0.9652	0.9652	**0.9652**	0.9826	0.9652	ND	ND	ND	0.9722	0.9722	0.9703	0.9815	0.9789	0.9792	1	1
SWS Sa10-1 2010 Poland	0.8903	0.8148	0.7948	0.8584	0.8906	0.815		0.9972	0.9910	**1**	0.9889	0.9698	1	0.9851	0.9779	0.9909	0.9727	0.9703	0.9636	0.9789	0.9583	0.9652	0.9652
SWS Sa10-5 2010 Poland	0.8903	0.8148	0.7948	0.8584	0.8906	0.815	0.9972		0.9910	**1**	0.9889	0.9698	1	0.9851	0.9779	0.9909	0.9727	0.9703	0.9636	0.9789	0.9583	0.9652	0.9652
SWS Sa10-6 2010 Poland	0.8903	0.8148	0.7948	0.8584	0.8906	0.815	0.9960	0.996		**1**	0.9820	0.9698	1	0.9851	0.9779	0.9909	0.9727	0.9703	0.9636	0.9789	0.9583	0.9652	0.9652
**SWS PL7663JH151 2017 Poland**	**0.9885**	**0.7954**	**0.7948**	**0.8584**	ND	**0.8237**	**0.8818**	**0.8818**	**0.8818**		**0.9826**	**0.9652**	**ND**	**ND**	**ND**	**0.9907**	**0.9722**	**0.9703**	**0.963**	**0.9789**	**0.9583**	**0.9652**	**0.9652**
SWSV mp70 2006 Switzerland	0.8208	0.8414	0.8399	0.7978	0.8168	0.8150	0.8278	0.8292	0.8277	**0.8040**		0.9704	1	0.9851	0.9779	0.9727	0.9727	0.9703	0.9818	0.9789	0.9583	0.9826	0.9826
SWS MSB94609 2000 Hungary	0.8337	0.8159	0.8062	0.8090	0.8346	0.8266	0.8105	0.8105	0.8105	**0.8329**	0.8101		0.9932	0.9776	0.9853	0.9727	0.9727	0.9703	0.9636	0.9789	0.9792	0.9652	0.9652
SWS MSB95463 1997 Hungary	0.8219	0.8263	ND	ND	0.8092	ND	0.8091	0.8091	0.8091	**ND**	0.7932	0.8250		0.9851	0.9779	ND	ND	ND	ND	ND	ND	ND	ND
SWS DGR18874 1982 Finland	0.7970	0.8114	ND	ND	0.7939	ND	0.8144	0.8144	0.8144	**ND**	0.7921	0.8020	0.7748		0.9627	ND	ND	ND	ND	ND	ND	ND	ND
SWS DGR18207 1982 Finland	0.7995	0.8088	ND	ND	0.7939	ND	0.8093	0.8093	0.8093	**ND**	0.8020	0.8068	0.7971	0.7896		ND	ND	ND	ND	ND	ND	ND	ND
SWS Beskydy415 2010 Czech	0.8333	0.8333	0.8241	0.8241	ND	0.8364	0.8333	0.8333	0.8333	**0.8277**	0.8212	0.8485	ND	ND	ND		0.9818	0.9802	0.9727	0.9895	0.9687	0.9722	0.9722
SWS Kosice260 2008 Slovakia	0.8242	0.8242	0.8241	0.7994	ND	0.8364	0.8152	0.8152	0.8152	**0.8154**	0.8030	0.8424	ND	ND	ND	0.8242		0.9901	0.9727	1	0.9792	0.9722	0.9722
SWS Koednitz08068 2007 Germany	0.8284	0.8053	0.7987	0.8119	ND	0.8317	0.8218	0.8218	0.8218	**0.8251**	0.7954	0.8053	ND	ND	ND	0.8020	0.8350		0.9703	0.9895	0.9687	0.9703	0.9703
SWS Horst092292 2009 Germany	0.8121	0.7970	0.7963	0.7685	ND	0.8210	0.7909	0.7909	0.7909	**0.8062**	0.8091	0.8394	ND	ND	ND	0.8061	0.8333	0.8251		0.9789	0.9583	0.9815	0.9815
SWS Goriska6193 2013 Slovenia	0.8014	0.8049	0.7979	0.8153	ND	0.8118	0.8258	0.8258	0.8258	**0.7875**	0.8049	0.8676	ND	ND	ND	0.8328	0.8188	0.8084	0.8153		0.9789	0.9789	0.9789
SWS NotrKras36290 2013 Slovenia	0.8125	0.7951	0.7951	0.8368	ND	0.8160	0.8299	0.8299	0.8299	**0.8125**	0.8160	0.8681	ND	ND	ND	0.8507	0.8299	0.8299	0.8056	0.8606		0.9792	0.9792
SWS Telet-300 2007 Russia	0.8213	0.8213	0.8208	0.7890	ND	0.8179	0.8156	0.8156	0.8156	**0.8184**	0.7983	0.7810	ND	ND	ND	0.7785	0.7754	0.7987	0.8031	0.8188	0.8125		1
SWS ParnayaSa1197 2008 Russia	0.8271	0.8386	0.8353	0.8121	ND	0.8092	0.8098	0.8098	0.8098	**0.8242**	0.8213	0.7925	ND	ND	ND	0.8123	0.8000	0.8185	0.7969	0.8014	0.8264	0.9164	

ID: not detected (sequence homologies of 10 nt or less were excluded from the analysis).

Supplemental Table S1. Pairwise Comparison of L-Segment Nucleotide Sequences of NVAV Strains

Amino Acid Sequence Similarity

	NVAV MSB95703 1999 Hungary	NVAV YA0067 2013 France	NVAV YA0088 2013 France	NVAV BENamurTE 2013 Belgium	NVAV BEGenappeTE 2013 Belgium	NVAV 1129 2010 Poland	NVAV 2086 2011 Poland	NVAV 2105 2011 Poland	NVAV 3328 2013 Poland	NVAV Te34 2013 Poland	NVAV PL7690JH204 2017 Poland	NVAV PL7691JH205 2017 Poland	NVAV PL7698JH212 2017 Poland	NVAV PL7706JH220 2015 Poland	NVAV PL7710JH224 2017 Poland	NVAV PL7712JH226 2012 Poland	NVAV PL7713JH227 2012 Poland	NVAV PL7714JH228 2012 Poland	NVAV PL7965LW001TA 2016 Ukraine	NVAV PL7970LW006TA 2016 Ukraine
NVAV MSB95703 1999 Hungary		0.9737	0.9774	0.9708	0.9708	0.9854	0.9847	0.9883	0.9744	0.9638	0.9658	0.9658	0.9655	0.9658	0.9658	0.9744	0.9573	0.9573	0.9720	0.9744
NVAV YA0067 2013 France	0.8365		0.9737	0.9624	0.9737	0.9699	0.9695	0.9699	0.9658	0.9699	0.9573	0.9573	0.9569	0.9573	0.9573	0.9658	0.9487	0.9487	0.9720	0.9744
NVAV YA0088 2013 France	0.8577	0.8477		0.9812	0.9774	0.9737	0.9733	0.9737	0.9658	0.9737	0.9573	0.9573	0.9569	0.9573	0.9573	0.9658	0.9487	0.9487	0.9533	0.9573
NVAV BENamurTE 2013 Belgium	0.8548	0.8215	0.8639		0.9801	0.9737	0.9733	0.9766	0.9658	0.9634	0.9573	0.9573	0.9569	0.9573	0.9573	0.9658	0.9487	0.9487	0.9626	0.9658
NVAV BEGenappeTE 2013 Belgium	0.8565	0.8365	0.8290	0.8778		0.9795	0.9809	0.9825	0.9829	0.9606	0.9744	0.9744	0.9741	0.9744	0.9744	0.9829	0.9658	0.9658	0.9720	0.9744
**NVAV 1129 2010 Poland**	0.8635	0.8325	0.8487	0.8694	0.8694		1	0.9971	1	0.9971	0.9915	0.9915	0.9914	0.9915	0.9915	1	0.9829	0.9829	0.9626	0.9658
**NVAV 2086 2011 Poland**	0.8668	0.8338	0.8541	0.8655	0.8503	**0.9645**		1	1	1	0.9915	0.9915	0.9914	0.9915	0.9915	1	0.9829	0.9829	0.9626	0.9658
**NVAV 2105 2011 Poland**	0.8713	0.8312	0.8475	0.8674	0.8674	**0.9688**	**0.9848**		1	1	0.9915	0.9915	0.9914	0.9915	0.9915	1	0.9829	0.9829	0.9626	0.9658
**NVAV 3328 2013 Poland**	0.8437	0.8551	0.8551	0.8494	0.8523	**0.9972**	**0.9659**	**0.9801**		1	0.9915	0.9915	0.9914	0.9915	0.9915	1	0.9829	0.9829	0.9626	0.9658
**NVAV Te34 2013 Poland**	0.8539	0.8365	0.8464	0.8602	0.8556	**0.9708**	**0.9632**	**0.9669**	**0.9716**		0.9915	0.9915	0.9914	0.9915	0.9915	1	0.9829	0.9829	0.9626	0.9658
**NVAV PL7690JH204 2017 Poland**	0.8470	0.8555	0.8555	0.8470	0.8470	**0.9632**	**0.9660**	**0.9575**	**0.9602**	**0.9603**		1	1	1	1	0.9915	0.9915	0.9915	0.9626	0.9573
**NVAV PL7691JH205 2017 Poland**	0.8442	0.8527	0.8584	0.8499	0.8442	**0.9603**	**0.9575**	**0.9547**	**0.9574**	**0.9575**	**0.9915**		1	1	1	0.9915	0.9915	0.9915	0.9626	0.9573
**NVAV PL7698JH212 2017 Poland**	0.8442	0.8584	0.8527	0.8499	0.8499	**0.9660**	**0.9632**	**0.9603**	**0.9631**	**0.9632**	**0.9972**	**0.9943**		1	1	0.9914	0.9914	0.9914	0.9626	0.9569
**NVAV PL7706JH220 2015 Poland**	0.8470	0.8555	0.8555	0.8470	0.8470	**0.9632**	**0.9660**	**0.9575**	**0.9602**	**0.9603**	**1**	**0.9915**	**0.9972**		1	0.9915	0.9915	0.9915	0.9626	0.9573
**NVAV PL7710JH224 2017 Poland**	0.8470	0.8555	0.8555	0.8470	0.8470	**0.9632**	**0.9660**	**0.9575**	**0.9602**	**0.9603**	**1**	**0.9915**	**0.9972**	**1**		0.9915	0.9915	0.9915	0.9626	0.9573
**NVAV PL7712JH226 2012 Poland**	0.8442	0.8555	0.8499	0.8499	0.8527	**1**	**0.9688**	**0.9830**	**0.9972**	**0.9688**	**0.9632**	**0.9603**	**0.9660**	**0.9632**	**0.9632**		0.9829	0.9829	0.9626	0.9658
**NVAV PL7713JH227 2012 Poland**	0.8414	0.8555	0.8442	0.8470	0.8442	**0.9632**	**0.9603**	**0.9575**	**0.9602**	**0.9547**	**0.9887**	**0.9858**	**0.9915**	**0.9887**	**0.9887**	**0.9632**		0.9829	0.9626	0.9573
**NVAV PL7714JH228 2012 Poland**	0.8414	0.8555	0.8442	0.8470	0.8442	**0.9632**	**0.9603**	**0.9575**	**0.9602**	**0.9547**	**0.9887**	**0.9858**	**0.9915**	**0.9887**	**0.9887**	**0.9632**	**0.9943**		0.9533	0.9487
**NVAV PL7965LW001TA 2016 Ukraine**	0.8395	0.8549	0.8395	0.8179	0.8179	0.8426	0.8519	0.8488	0.8457	0.8488	0.8580	0.8488	0.8549	0.8580	0.8580	0.8426	0.8488	0.8488		**1**
**NVAV PL7970LW006TA 2016 Ukraine**	0.8442	0.8555	0.8414	0.8300	0.8300	0.8555	0.8640	0.8555	0.8580	0.8669	0.8669	0.8584	0.8640	0.8669	0.8669	0.8555	0.8584	0.8584	**0.9815**	
