# Supplementary material for: Phylogeny of Shrew- and Mole-Borne Hantaviruses in Poland and Ukraine
Source: Viruses. 2023 Mar 29;15(4):881. doi: 10.3390/v15040881 (PMC10145205; doi:10.3390/v15040881)
Supplement: Supplementary file 1 [file viruses-15-00881-s001.zip › Table S2.pdf]

Supplemental Table S2. GenBank Accession Numbers of Taxa for S and L trees

Virus	Strain	Year	Capture Site	Country	S	L
SWSV	1107	2010	Boginia	Poland	JX990921	JX990936
SWSV	2059	2011	Chmiel	Poland	KM394269	JX990941
SWSV	2063	2011	Chmiel	Poland	KM394270	JX990942
SWSV	2049	2011	Huta Dłutowska	Poland	KM394271	JX990945
SWSV	3334	2013	Kurowice	Poland	KM434198	KM394263
SWSV	3343	2013	Kurowice	Poland	KM434199	KM394265
SWSV	Sa10-1	2010	Osobowice	Poland	MK402016	MK402015
SWSV	Sa10-5	2010	Osobowice	Poland	MK402019	MK402017
SWSV	Sa10-6	2010	Osobowice	Poland	MK402022	MK402020
SWSV	mp70	2006	Seewis	Switzerland	EF636024	EF636026
SWSV	MSB94609	2000	Zala	Hungary	GQ293138	GQ293123
SWSV	MSB95463	1997	Gyor-Sopron-Moson	Hungary	GQ293136	GQ293116
SWSV	DGR18874	1982	Oulun Lääni	Finland	GQ293132	GQ293111
SWSV	DGR18207	1982	Lappi	Finland	GQ293126	GQ293103
SWSV	Beskydy415	2010	Beskydy	Czech Rep	JQ425306	JQ425341
SWSV	Košice260	2008	Košice	Slovakia	JQ425272	JQ425317
SWSV	Ködnitz08068	2007	Ködnitz	Germany	JQ425260	JQ425309
SWSV	Horst092292	2009	Horst	Germany	JQ425265	JQ425311
SWSV	Goriska_61_9	2013	Goriska	Slovenia	KF060930	KF060925
SWSV	Notr-Kras_362_90	2013	Notr-Kras	Slovenia	KF060931	KF060924
SWSV	Telet-Sa300	2007	Teletskoye Lake	Russia	MN815797	EU424334
SWSV	Parnaya-Sa1197	2008	Parnaya	Russia	MG279214	MG279210
BOGV	2074	2011	Boginia	Poland		JX990965
BOGV	2073	2011	Huta Dłutowska	Poland		JX990964
BOGV	2177	2012	Kurowice	Poland		KC537795
BOGV	LaihiaL	2005	Laihia	Finland		KJ136642
NVAV	2086	2011	Brosinin	Poland	KF515970	JX990963
NVAV	1129	2010	Huta Dłutowska	Poland	JX990922	JX990946
NVAV	2105	2011	Huta Dłutowska	Poland	JX990935	JX990962
NVAV	3328	2013	Huta Dłutowska	Poland	KM403429	KM394244
NVAV	MSB95703	1999	Nova	Hungary	FJ539168	FJ593498
NVAV	YA0067	2013	Ozoir-la-Ferrière	France	KF010576	KF010544
NVAV	YA0088	2013	Beauvais	France	KF010570	KF010533
NVAV	BE/Vieux-Genappe/TE/2013/2	2013	Vieux-Genappe	Belgium	KY780086	KY780088
NVAV	BE/Namur/TE/2013/1	2013	Namur	Belgium	KT004445	KT004447
BRGV	DE/Wandlitz/TE/2013/1	2013	Wandlitz	Germany	MF683844	MF683846
BRGV	BE/Vieux-Genappe/TE/2013/1	2013	Vieux-Genappe	Belgium	KX551960	KX551962
ALTV	ALT302	2007	Teletskoye Lake	Russia	MK340902	MT648514
ALTV	Parabel-Sa44	2019	Parabel	Russia	MT560057	MN815789
ALTV	MSB95363	1997	Nógrád	Hungary	GQ293127	GQ293104
ALTV	Uurainen63L	2004	Uurainen	Finland		KJ136623
ALTV	Smin1108	2010	Chmiel	Poland		MN244248
LENV	MSB146482	2010	Lena River	Russia	KM361043	KM361055
LENV	Parnaya-Sc1217	2008	Parnaya	Russia	MW505551	MW505552
LENV	Khekhtsir-Sc67	2008	Khekhtsir	Russia	MH499470	MH499472

GenBank Accession numbers of newfound hantavirus sequences from Poland and Ukraine

Hantavirus	Strain	Year	Capture Site	Country	S GenBank	nt	L GenBank	nt
SWSV	PL7663JH151	2017	Boginia	Poland			OQ341654	347
ALTV	PL7814LR56	2004	Białowieża Forest	Poland			OQ341663	353
NVAV	PL7690JH204	2017	Huta Dłutowska	Poland			OQ341655	353
NVAV	PL7691JH205	2017	Huta Dłutowska	Poland			OQ341656	353
NVAV	PL7698JH212	2017	Huta Dłutowska	Poland			OQ341657	353
NVAV	PL7706JH220	2015	Huta Dłutowska	Poland			OQ341658	353
NVAV	PL7710JH224	2017	Huta Dłutowska	Poland			OQ341659	353
NVAV	PL7712JH226	2012	Huta Dłutowska	Poland	OQ352297	707	OQ341660	353
NVAV	PL7713JH227	2012	Huta Dłutowska	Poland			OQ341661	353
NVAV	PL7714JH228	2012	Huta Dłutowska	Poland			OQ341662	353
NVAV	PL7965LW001TA	2016	Lviv	Ukraine			OQ341664	324
NVAV	PL7970LW006TA	2016	Lviv	Ukraine	OQ352298	698	OQ341665	353

Representative Hantaviruses Harbored by Rodents, Shrews and Moles

Hantavirus	Strain	Country	S	L
TPMV	VRC66412	India	AY526097	EU001330
MJNV	Cl05-11	Korea	EF641804	EF641806
ULUV	FMNH158302	Tanzania	JX193695	JX193697
KMJV	FMNH174124	Tanzania	JX193698	JX193700
CBNV	CBN-3	Vietnam	EF543524	EF543525
JJUV	SH42	Korea	HQ663933	HQ663935
AZGV	KBM15	Côte d'Ivoire	JF276226	JF276228
BOWV	VN1512	Guinea	KC631782	KC631784
TGNV	Tan826	Guinea	EF050455	EF050454
ARRV	MSB73418	United States	EF650086	EF619961
YKSV	Si-210	China	JX465423	JX465389
JMSV	MSB144475	United States	FJ593499	FJ593501
KKMV	MSB148794	Russia	GQ306148	GQ306150
ASIV	CZ/Drahany/420/2010/Sm	Czech Republic	KC880342	KC880348
ASAV	N10	Japan	EU929072	EU929078
OXBV	Ng1453	United States	FJ5339166	FJ593497
RKPV	MSB57412	United States	HM015223	HM015221
TIGV	ET2121	Ethiopia	KU934010	KU934008
SNV	NMH10	United States	NC_005216	NC_005217
ANDV	Chile9717869	Chile	AF291702	AF291704
PHV	PH-1	United States	Z49098	EF646763
TULV	M5302v	Czech Republic	NC_005227	NC_005226
PUUV	Sotkamo	Finland	NC_005224	NC_005225
SANGV	SA14	Guinea	JQ082300	JQ082302
SOOV	SOO-1	Korea	AY675349	DQ056292
DOBV/BGDV	Greece	Greece	NC_005233	NC_005235
HTNV	76-118	Korea	NC_005218	NC_005222
SEOV	80-39	Korea	NC_005236	NC_005238
